# Injury induced activation of extracellular signal-regulated kinase (ERK) in the rat rostral ventromedial medulla (RVM) is age dependant and requires the lamina I projection pathway

**DOI:** 10.1186/1744-8069-6-54

**Published:** 2010-09-14

**Authors:** Sandrine M Géranton, Keri K Tochiki, Winnie WY Chiu, Sarah A Stuart, Stephen P Hunt

**Affiliations:** 1 Department of Cell and Developmental Biology, University College London, WC1E 6BT, UK

## Abstract

Descending controls originating in part from the rostral ventromedial medulla (RVM) regulate the excitability of dorsal horn neurons and maintain peripheral pain states. Activation of extracellular signal regulated kinase (ERK) in RVM neurons has been shown following peripheral inflammation and is involved in generating the accompanying inflammatory hyperalgesia. Here, we show that spared nerve injury (SNI), a model of neuropathic pain, results in an increase in ERK activity in RVM neurons of adult rats 3 and 8 days following surgery. We carried out two experimental procedures to demonstrate that this increase in ERK activation was related to the increased mechanical sensitivity associated with SNI. First, we showed that lesions of the lamina I/III ascending pathway from the dorsal horn attenuated both mechanical hyperalgesia and ERK activation in the RVM. Second, we performed SNI in P10 rats. At this age, SNI did not result in mechanical hypersensitivity, as previously shown, and did not activate ERK in the RVM. Finally, the percentage of pERK expressing neurones that were also serotonergic was always around 60%, independent of pain state and age, indicating an important role for serotonin in descending controls of pain states.

## Background

The rostral ventromedial medulla (RVM) plays an essential role in the regulation of spinal cord nociceptive activity and pain transmission [1-5]. The RVM, inclusive of the nucleus raphe magnus, nucleus reticularis gigantocellularis and ventral nucleus reticularis gigantocellularis, receives projections from the periaqueductal grey and, in the adult rat, [5,6] activation of separate populations of RVM neurons generates either descending inhibition or facilitation [7-10]. It has been shown that high intensity stimulation within the RVM inhibited spinal nociception while low intensity stimulation was facilitatory [6,11]. Finally, inactivation of the RVM with lidocaine or excitatory block with the glutamate receptor antagonist kynurenate lead to attenuation of mechanical allodynia caused by inflammation or peripheral nerve ligation respectively [12,13].

The Mitogen Activated Protein (MAP) kinases are known to play an essential role in the regulation of neuronal plasticity *via *post-translational and transcriptional modifications. An increase in spinal extracellular signal-related kinase (ERK) activity has been implicated in the induction and/or maintenance of pain states following noxious stimulation of the periphery [14-16]. Phospho-ERK (pERK) was also up-regulated in the RVM following peripheral inflammation induced by Complete Freund's Adjuvant (CFA)injected into the hind paw [17] and inhibition of ERK signaling within the RVM somewhat reduced thermal hyperalgesia at 24 h (but not 7 h) after inflammation [17,18].Although up-regulation of pERK after digit amputation has been described in the anterior cingulate cortex [19], the link between ERK activity in the RVM and increased pain sensitivity in models of neuropathic pain remains to be elucidated.

Descending controls from the RVM are indirectly modulated by activity in ascending pathways originating in lamina I/III projection neurons of the dorsal horn. Selective ablation of these ascending projections leads to significant attenuation of inflammation-induced hyperalgesia and neuropathic pain [20,21]. We have therefore investigated the influence of lamina I/III projection neurones on ERK activation in the RVM in both neuropathic and inflammatory pain models. We found that ERK activation in the RVM is associated with mechanical hyperalgesia but that both the pain states and pERK expression are reduced by lamina I ablation.

Finally, it has been shown that young animals do not develop neuropathic pain for at least 14 days post-surgery [22]. Therefore, if ERK activation in the RVM plays a crucial role in the maintenance of neuropathic pain states, we hypothesized that ERK would not be activated in the RVM of the young animal following surgery. We found that this was indeed the case implying that activation of ERK in the RVM is correlated with the development of neuropathic pain states.

## Methods

### Subjects

All procedures complied with the UK Animals (Scientific Procedures) Act 1986. Male Sprague Dawley rats (P10 and 170-200 g; Central Biological Services, University College London, UK) were used for all experiments. Animals were kept in their home cages at 21°C and 55% relative humidity with a light-dark cycle of 12 h (lights on at 08:00 h). Food and water were provided *ad libitum*. All efforts were made to minimise animal suffering and to reduce the number of animals used.

### Antibodies and drugs

Anti- phospho p44/42 MAP kinase (ERK 1/2) (pERK; Thr 202/Tyr 204) was obtained from Cell Signaling Technology (Danvers, MA). Anti-tryptophan hydroxylase (anti-TPH) was from Sigma (Poole, UK) and anti-NK1 antibody was a gift from S. Vigna. Finally [Sar9,Met(O2)11]substance P coupled to saporin (SSP-SAP) from Advanced Targeting Systems (San Diego, CA) [23].

### Ablation of lamina I projection neurons by intrathecal injections of SSP-SAP

Rats under isoflurane anaesthesia (1.5-2% isoflurane combined with 100% O2 (1l/min)) were placed in a stereotaxic frame and a small incision was made in the atlanto-occipital membrane. A cannula was inserted into the subarachnoïd space, terminating in L4-5region. Animals received either 10 μl (100 ng) of SSP-SAP in saline or 10 μl of saline [23]. The cannula was then withdrawn and the wound closed with sutures. Animals were kept in their home cage for 4 weeks until further experiment. The efficacy of SSP-SAP ablation was assessed following NK1 immunohistochemistry.

### Spared nerve injury surgery

The spared nerve injury (SNI) was performed as described [24]. Under 2% isoflurane anaesthesia the biceps femoris muscle was exposed and sectioned to expose the sciatic nerve and its three terminal branches: the sural, common peroneal and tibial nerves. The common peroneal and tibial nerves were tightly ligated with 5.0 silk and sectioned distal to the ligation. Care was taken to avoid touching or stretching the spared sural nerve. For sham surgery, the sciatic nerve was exposed as described above but no contact was made with the nerve.

### Behavioural assay

*Von-Frey test: *mechanical sensitivity was assessed using the Von-Frey test as described [25]. A series of calibrated Von-Frey hairs were applied to the lateral plantar surface of the paw, in ascending order. The threshold was taken as the lowest force required to elicit a response to one of five repetitive stimuli.

### Immunohistochemistry

For immunohistochemistry, rats were deeply anaesthetized with pentobarbital and perfused transcardially as described [26]. The spinal cord and brain were dissected out, post-fixed in the same PFA 4% solution for 2 h and transferred into a 30% sucrose solution in PB containing 0.01% azide, for a minimum of 24 h. Tissue was cut on a freezing microtome at 40 μm. For pERK staining, free floating tissue sections were blocked for 1 h in 3% normal goat serum and 0.3% triton X-100 in 0.1 M PB. Tissue was then left to incubate in anti-pERK (1:250) in TTBS for 3 days at 4°C. Biotinylated secondary antibody was subsequently applied at a dilution of 1:400 in TTBS for 1.5 h, followed by 30 min in avidin-biotin peroxidase complex solution diluted to 1:250 of each Vectastain A and Vectastain B solutions. A further tyramide signal amplification step was performed at a dilution of 1:75 biotinylated tyramide in diluent (PerkinElmer LAS, Boston, MA) for 7 min. Sections were finally incubated with FITC (Vector Laboratories, Burlingame, CA), 1:600 in TTBS for 2 h in a dark box. When double label with TPH was required, the tissue was then placed in anti-TPH (1:1000) overnight in the dark at room temperature. The following day, the sections were incubated for 2 h in Alexa Fluor 594 conjugated secondary antibody (Invitrogen, Eugene, OR). After washing, sections were mounted onto gelatinised slides. Once dry, slides were coverslipped with Fluoromount (Sigma, UK) to minimise any fading of fluorescence. Controls for double-staining included omission of the second primary antibody. For NK1 receptor staining, sections were incubated overnight (1:5,000) at room temperature. On the following day, the appropriate biotinylated antibody (1:500, 2 h) was applied followed by Cy3-streptavidin (1:4,000, 45 min). After final washes, sections were mounted as described above.

### Cell counting

**Figure 1:**
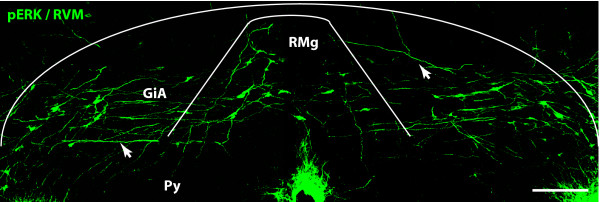
**pERK expression in the RVM after SNI surgery**. RMg: nucleus raphe magnus; GiA: nucleus reticularis gigantocellularis pars alpha; Py: pyramidal tract. Scale bar, 200 μm.

The number of immunopositive cells expressing pERK were counted bilaterally within the RVM using the anatomical limits described by Imbe. et al. [17,18] (Fig. 1). TPH was used as a marker to label serotonergic neurones to define the RVM. Immunopositive cells were counted and summed in the 5 RVM sections with the most pERK labeling for each animal. The mean across animals was used for further statistical analysis. Counts of ERK activation within serotonergic neurons were also done bilaterally within in the RVM.

### Statistical data analysis

All statistical tests were performed in SPSS PC+. To analyse behavioural data, repeated measures ANOVA, followed by Tukey or Bonferroni post-hoc analysis where appropriate (SPSS+), was used. The Greenhouse-Geisser 'ε' correction was applied to compensate for any violation of sphericity. Data were normalized by logarithmic (log2) transformation. For pERK, pERK and TPH and NK1 receptor positive cells counting, the number of labeled cells was analysed by univariate analysis with relevant post-hoc analysis. Data were always analysed as presented in the figures (raw data or log2 transformed).

## Results

### Phosphorylation of ERK in the RVM increases with mechanical hypersensitivity in a model of neuropathic pain, both in the induction and the maintenance phase

Adult rats were divided into three groups. Animals in the first group were dedicated to behaviour testing: after establishing mechanical thresholds using Von Frey filaments, adult rats underwent SNI or sham surgery and mechanical thresholds were measured for another 7 days. The other 2 groups of animals were dedicated to histochemical analysis of pERK expression in the RVM: animals underwent SNI or sham surgery and were perfused on day 3 or on day 8 post-surgery.

**Figure 2:**
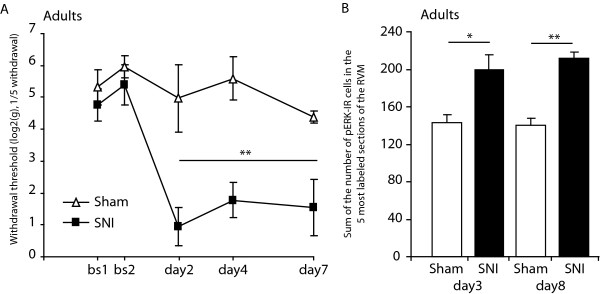
**Phosphorylation of ERK in the RVM increases with mechanical sensitivity in a model of neuropathic pain**. Effects of SNI or sham surgery in adult rats (N = 4-5 in each group) on: A/Mechanical withdrawal thresholds measured by Von Frey testing of the hind paw of the operated limb; and B/pERK expression in the RVM 3 and 8 days after surgery. Adult rats developed mechanical hypersensitivity and showed increase ERK activation in the RVM at 3 and 8 days post-SNI. Data show mean ± SEM. * *P *< 0.05, ** *P *< 0.01.

As expected, adult rats having undergone SNI showed increased mechanical sensitivity from day 2 to day 7 post- surgery (Fig.2A; F_1,8 _= 15.9, *P *< 0.01, SNI *vs *sham, day 2 today 7). Moreover, the number of pERK positive cells within the RVM was greater in SNI animals when compared with sham animals both on day 3 (Fig. 2B; 200 ± 16 *vs *143 ± 8 respectively, *P *< 0.05) and day 8 post-surgery (Fig. 2B; 212 ± 6 *vs *140 ± 7 respectively, *P *< 0.01).

### Ablation of NK1 receptor expressing neurones in the dorsal horn reduces the expression of SNI induced pERK in the RVM

**Figure 3:**
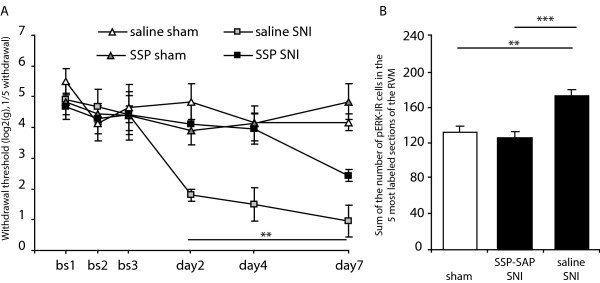
**Ablation of NK1 receptor expressing neurones in the dorsal horn reduces SNI induced decrease in mechanical threshold and ERK phosphorylation in the RVM**. Effect of SSP-SAP or saline intrathecal injections on: A/mechanical threshold following SNI surgery measured by Von Frey testing and B/SNI induced pERK expression in the RVM 8 days after SNI surgery. SSP-SAP treatment significantly attenuated both the SNI induced hyperalgesia and the SNI induced ERK activation in the RVM. N = 3-5 in each group. Data show mean ± SEM. * *P *< 0.05, ** *P *< 0.01.

We investigated the role of lamina I projection neurones in the increase in mechanical sensitivity seen in SNI rats and the associated activation of ERK in the RVM. Animals received an intrathecal injection of SSP-SAP or saline and 4 weeks later underwent SNI or sham surgery (*ie*. animals were organised in 4 groups: saline sham, saline SNI, SSP-SAP sham and SSP-SAP SNI). Behavioural testing began the day after surgery and continued until day 7 post-surgery. On day 8 post-surgery, animals were perfused for pERK immunoreactivity analysis. Animals that underwent sham surgery showed no decrease in mechanical sensitivity until at least day 7 (Fig. 3A). Mechanical thresholds of animals having received saline SNI treatment significantly dropped from day 2 post-surgery and remained decreased until day 7 (Fig. 3A). However, when animals received an SSP-SAP injection prior to the SNI surgery, there was no decrease in mechanical sensitivity on day 2 and day 4 of testing and at day 7 their mechanical threshold was still significantly higher than that of the saline pre-treated group (Fig. 3A; F_1,8 _= 16.7, *P *< 0.01, post-hoc SSP-SAP SNI *vs *saline SNI). When we counted the number of pERK expressing cells within the RVM day 8 post SNI surgery, there was no difference between the 2 sham operated groups and results were pooled together (Fig. 3B; 134 ± 7).There was a significant increase in pERK expression when animals had received saline SNI treatment, both compared to SSP-SAP SNI and sham animals (Fig. 3B; 174 ± 7 *vs *127 ± 7, *P *< 0.001, and 174 ± 7 *vs *134 ± 7, *P *< 0.01, saline SNI *vs *SSP-SAP SNI and saline SNI *vs *sham respectively).

### Intrathecal injection of SSP-SAP reduces NK1 receptor expression in the superficial dorsal horn

**Figure 4:**
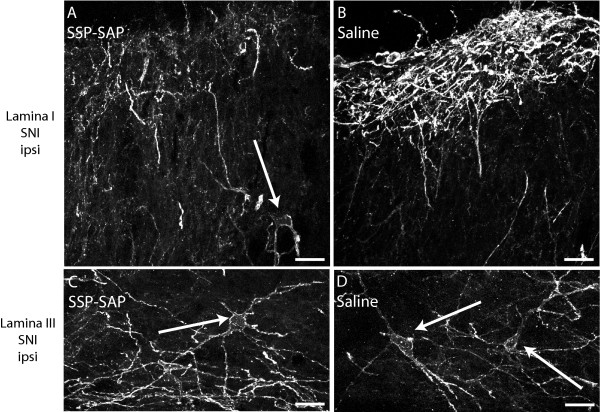
**Intrathecal injection of SSP-SAP reduces NK1 receptor expression in the superficial dorsal horn**. Effect of SSP-SAP or saline intrathecal injections on NK1 receptor expression in the superficial dorsal horn. A, B/Confocal images of NK1 receptor immunohistochemistry in lamina I of SNI animals after SSP-SAP or saline treatment. C,D/Confocal images of NK1 receptor immunohistochemistry in lamina III of SNI animals after SSP-SAP or saline treatment. All images taken from the dorsal horn. contralateral to SNI. Arrows show cell bodies within lamina III. Scale bar, 100 μm.

To confirm that NK1 receptor immunoreactivity was reduced in the dorsal horn following SSP-SAP treatment we processed tissue from each of the rats described in the experiment above. Immunostaining for the NK1 receptor showed a marked reduction inimmunofluorescence when animals had received SSP-SAP treatment (Fig. 4A-D).

### P10 rats do not develop neuropathic pain and do not show any increase in pERK expression in the RVM following SNI

**Figure 5:**
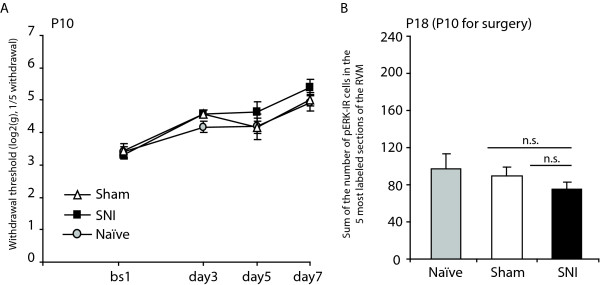
**P10 rats do not develop neuropathic pain and do not show any increase in pERK expression in the RVM following SNI**. Effects of SNI or sham surgery in P10 rats (N = 3 in each group), on: A/Mechanical withdrawal thresholds measured by Von Frey testing of the hind paw of the operated limb; and B/pERK expression in the RVM, 8 days after surgery. P10 rats never developed mechanical hypersensitivity and there was no difference in pERK expression in their RVM. Data show mean ± SEM.

After establishing mechanical thresholds by Von Frey testing, P10 rats underwent SNI, sham surgery or were put under isoflurane anaesthesia. Mechanical thresholds were measured for another 7 days and on day 8 animals were perfused for histochemical analysis of pERK expression in the RVM. P10 animals have been shown not to develop neuropathic pain and indeed we found no difference in mechanical threshold between naïve, sham and SNI until day 7 following surgery (Fig. 5A). Moreover, when we analysed pERK staining within the RVM for these animals 8 days following surgery (P18 animals) we did not find any differences between the 3 groups of animals (Fig. 5B; number of pERK labelled cells within the RVM: 97 ± 16, 90 ± 9 and 75 ± 8, naïve, sham and SNI respectively).

### ERK activation in the RVM is mainly within 5HT expressing neurones and the proportion of 5HT/pERK positive neurons does not change with pain state or age

**Figure 6:**
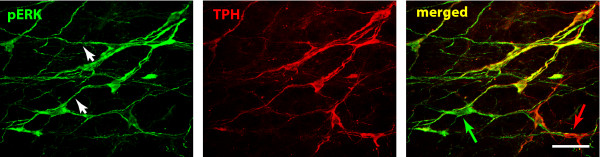
**60% of pERK expressing neurons in the RVM are also serotonergic**. Confocal images of ERK activation in the RVM 8 days post-SNI surgery. Colocalization of ERK expressing neurons (*green) *and TPH expressing neurones (*red*). The single staining for each antibody and the merged image are shown from left to right and double staining appears in *yellow*. Notice the extensive staining of dendrites (arrow heads). Arrows show non double labeled neurons. Scale bar, 200 μm.

There was no difference in the number of pERK positive neurons in the serotonergic population between sham and SNI animals in P18 rats, 52% ± 5% and 56% ± 4% of pERK expressing neurones were serotonergic, respectively. The proportion of serotonergic pERK expressing neurones also remained constant in adult rats after SNI or sham surgery, with saline or SSP-SAP pre-treatment: saline sham: 60% ± 1%, SSP-SAP sham: 61% ± 3%, saline SNI: 57% ± 4% and SSP-SAP SNI: 62% ± 3%. Fig. 6 shows double neurones labelled for pERK and 5HT in the RVM of adult rats following SNI.

## Discussion

Here, we show that ERK is activated within the adult RVM during both the induction and the maintenance phase of a neuropathic pain state. This activation is correlated with the mechanical hypersensitivity that follows tissue injury. Two experiments strongly implied that pERK was causally related to increased mechanical sensitivity. First, ablation of lamina I projection neurones attenuated the activation of ERK in the RVM after SNI surgery, as well as the induced mechanical hypersensitivity. Second, SNI in neonatal rats did not activate ERK in the RVM and there was no mechanical hyperalgesia.

In the present study, we investigated the activation of ERK within the RVM after tissue injury at different time points corresponding to induction and maintenance of the pain state. We investigated ERK activation in the adult RVM 3 and 8 days following SNI and found that pERK was elevated compared to SHAM to the same extent at both time points. Indeed, both at 3 and 8 days following SNI surgery, the number of pERK expressing neurones was 1.5 times higher than that in sham. This indicated that ERK remains activated within the RVM and may be important in maintaining the pain state. In our study, 8 days post SNI surgery, pERK expression within the RVM was limited to neurons and no glial activation was seen. p38, another MAPK involved in the maintenance of pain states, was found in microglia in the RVM 3 h after carrageenan injection into the hindpaw [27]. However, we did not find pERK within microglia or astrocytes, 8 days post SNI in the RVM (data not shown).

Interestingly, we also found that the proportion of pERK neurones labelled with TPH did not change after nerve injury although the total number of labelled neurons increased with treatment. In contrast, others have reported that the proportion of pERK neurons positive for 5HT increased from 30% to 60% 7 h after CFA injection (3% and 20% of the 5HT positive neurones respectively) [17]. However, it is important to notice that in this later study control animals were completely naïve (*i.e*. had receive no injection and were not put under anaesthesia) whereas in the present study control animals always underwent a sham operation.

Nevertheless, our results strongly imply that 5HT-positive RVM neurons are recruited during inflammatory and neuropathic pain states although this is always alongside a similar proportion of non-5HT positive neurons. Previous studies have indeed indicated that both RVM 5HT-dependent and -independent mechanisms regulate dorsal horn sensitivity and specific ablation of RVM 5HT neurons has been shown to modulate both electrical stimulation and BDNF induced hyperalgesia but not opiate induced analgesia [28]. RVM cells have previously been classified into 3 groups: ON-, OFF- and NEUTRAL- cells and it is believed that ON- and OFF- cells are recruited to engage respectively descending facilitation and inhibition from the brainstem [8,29,30]. Although only NEUTRAL- cells have been reported to contain 5HT [31], ON- and OFF cells have been shown to receive a strong serotonergic innervation, indicating that serotonin could both directly and indirectly control pain processing at the brainstem and spinal level [32,33]. Also, some serotonergic neurons express the mu opiate receptor [34] which has previously been suggested to be characteristic of non-5HT ON- cells. Specific ablation of RVM mu receptor expressing neurons by dermorphin-saporin conjugates alleviated pain states and would be expected to lesion both subsets of 5HT and non-5HT containing ON- cells. Descending 5HT projections from the RVM have been shown to contribute to the maintenance of chronic pain states at the 5HT3 receptor expressed by dorsal horn and primary afferent neurons [35-37]. Finally, there is evidence to suggest that NEUTRAL- cells (and therefore serotonergic neurons) can be recruited to become ON- or OFF- cells in persistent pain states [38] reinforcing the serotonergic contribution to descending modulation.

The role of pERK activation in RVM at 3 days post SNI is not clear. Previous results have shown that RVM is essential for the maintenance but not induction of mechanical hyperalgesia following peripheral nerve manipulations [39]. It seems likely that primary afferent activity following SNI is of particular importance in inducing the neuropathic pain state but that maintenance requires pERK activity in RVM neurons. However, given the high basal levels of pERK reported here, it is unlikely that direct injections of ERK inhibitors into the RVM would resolve this issue.

To test the hypothesis that ERK phosphorylation in the RVM is correlated with increased mechanical sensitivity, we investigated the influence of lesions of lamina I/III projection neurons with SSP-SAP. Previous research has shown that ablation of lamina I/III projection neurons attenuated most inflammatory and neuropathic pain states and that both induction and maintenance phases are reduced [20]. It was also shown that the immediate-early gene c-fos was up-regulated in the RVM following formalin injection into the hind paw and that this was reduced by lamina I/III lesions [36]. We therefore hypothesized that the loss of mechanical hypersensitivity in neuropathic pain states following ablation of lamina I/III projection neurons would be accompanied by reduced ERK activation in the RVM. Indeed, we found that ablation of lamina I/III projection neurons substantially reduced the activation of ERK in RVM neurons 8 days post surgery.

In a second set of experiments, we tested, in rat pups, the hypothesis that ERK phosphorylation in the RVM is correlated with hyperalgesia. Neonates do not develop mechanical hypersensitivity following the SNI procedure [22]. We therefore postulated that if ERK activation in the RVM and neuropathic sensitivity were linked, we would not see increased levels of pERK in the RVM of neonates that had undergone SNI. This was the case and there could be a number of reasons for this. First, it might be that the lamina I/III projection pathway is itself non-functional at birth. However, using c-fos histochemistry it has been possible to show that the lamina I/III-parabrachial nucleus projection neurones pathway is intact and functional in newborn rats. Dorsal horn neurones are responsive to noxious stimulation at P3 and capable of activating neurons within the parabrachial nucleus [40]. Therefore the absence of ERK activation within the RVM of neonates following SNI surgery is unlikely to be explained by the developmental state of ascending pathways. However, lamina I/III projection neurons are not thought to project directly to the RVM but through intermediate targets such as the PAG. It seems likely that the changing postnatal influence of the RVM in spinal nociception and the late development of the PAG-RVM connection [11,41] may account for the failure of SNI to promote mechanical hyperalgesia in neonates following peripheral nerve injury. This later point is of importance when considering the route by which RVM neurons are activated. One suggestion is through a connection between the PAG and the RVM known to release brain-derived neurotrophic factor (BDNF). Microinjection of BDNF into the RVM facilitates nociception. This action of BDNF is dependent on NMDA receptors and both inflammatory hyperalgesia and neuropathic pain can be inhibited by RVM injections of NMDA receptor antagonists [42,43]. BDNF has been shown in other systems to activate ERK [44] and a role of released BDNF in the activation of ERK in the RVM seems likely.

## Conclusions

In summary, we show that ERK activation in the RVM is correlated with increased mechanical hypersensitivity in a neuropathic pain model and is mediated by the lamina I/III dorsal horn projection pathway. We also show that there is a contribution of 5HT neurons to this process but always in concert with the activation of significant numbers of non-serotonergic RVM neurons.

## Abbreviations

**SNI**: Spared Nerve Injury; **ERK**: Extracellular Signal Regulated Kinase; **RVM**: Rostral Ventromedial Medulla.

## Competing interests

The authors declare that they have no competing interests.

## Authors' contributions

SMG and SPH conceived, designed and performed the experiments, analysed the data and wrote the manuscript. KKT, WWY and SAS performed some experiments and KKT contributed to the writing of the manuscript. All authors read and approved the final manuscript.
